# Can criminology sway the public? How empirical findings about deterrence affect public punishment preferences

**DOI:** 10.1186/s40163-024-00240-8

**Published:** 2024-12-18

**Authors:** Brendan Rose, Malouke Esra Kuiper, Chris Reinders Folmer, Benjamin van Rooij

**Affiliations:** 1 Center for Law and Behavior, Amsterdam Law School, University of Amsterdam, Amsterdam, The Netherlands; 2 Erasmus School of Law, Erasmus University Rotterdam, Rotterdam, The Netherlands; 3 School of Law, University of California, Irvine, CA USA

## Abstract

**Background setting:**

Punitive approaches to deter offending remain popular despite limited evidence of their effectiveness. This study investigated what effect presenting empirical criminological findings about the effectiveness of deterrence to a general public has on their punishment preferences. It builds on earlier research showing that such presentation reduces the public’s inclination towards strict punishment. The present study extended this research by exploring whether the impact of scientific evidence on public punishment preferences is affected by crime severity and by exploring cognitive and psychological factors that may underpin this relationship.

**Methods:**

Using a vignette study paradigm, a general public sample of 330 participants were asked to make hypothetical punishment decisions to reduce crime (whether or not to double sentences) for one of three crime types that varied in severity. For each crime type, half of participants were additionally provided with a summary of research on the deterrent effect of punitive policy measures.

**Results:**

Presenting scientific evidence reduced participants' preferences for stronger punishment and that this effect remained consistent regardless of crime severity—ranging from burglary to homicide. In addition, we did not find evidence that difference in individuals’ cognitive style, negative emotional reactions, perceptions about seriousness, or beliefs about redeemability moderated or mediated this relationship.

**Conclusions:**

This study provides compelling findings that further clarify the circumstances required for scientific evidence to be successfully disseminated to a general public to bring their punishment preferences more in line with the state of empirical science.

**Supplementary Information:**

The online version contains supplementary material available at 10.1186/s40163-024-00240-8.

## Introduction

There is limited evidence that tough-on-crime criminal justice policy, which seeks to deter crime via stronger punishment—such as increasing the length of prison sentences, effectively deters violent crime (Cullen et al., 2011; Nagin, 2013a; Piquero et al., 2011). Yet, these punitive criminal justice policies remain popular (Dunbar, 2022). Unfortunately, in these cases the large body of criminological scientific evidence has failed to shape public policy (Loughran, 2019; Nelken & Hamilton, 2022), resulting in continuing ineffective crime control strategies that come at high social and financial cost.

In democratic states, public opinion plays a major role in such tough-on-crime criminal justice policies through voting in elections, public referendums and through politicians drawing on public opinion to justify punitive criminal justice policies (Grimmelikhuijsen & van den Bos, 2021; Pickett, 2019; Shi, 2021). As such, the general public is a vital target to increase the influence of criminological evidence in public policy. Generally, studies have shown that public knowledge about criminal justice is limited (Pickett, 2019). Specifically, the general public is likely not familiar with criminological findings about deterrence. While in other fields of social science, such as social psychology or behavioral economics, popular books and magazines exist that have popularized key theories and empirical findings (e.g., Kahneman, 2011; Thaler & Sunstein, 2008), there is far less public dissemination in criminology. Moreover, as argued by Garland (2021), criminologists have had limited success entrenching their expertise within public policy discourse. Therefore, it may well be that the general public retain punitive attitudes because they do not know about the lack of scientific evidence for the deterrent effect of punishment for violent crime—and because criminologists have been, so far, comparatively unsuccessful at finding ways to communicate scientific evidence in ways to impact the opinions and decisions of the public. A crucial question therefore is how presenting the general public with empirical criminological evidence could influence their preferences for stronger punishment. Would citizens display more evidence-aligned punishment preferences when they learn that there is no conclusive evidence that these will act as an effective deterrent against violent crime (Dölling et al., 2009; Nagin, 2013a)? The present research seeks to answer this question.

### How criminological science may shape punishment preferences

Existing studies on public punitiveness have largely looked at what may explain variation in punitive attitudes, and in particular what may explain public preferences for punitive responses to crime. Public punitiveness—the public’s overall support/favorability towards harsher punishments—is a complicated area, which has shown to be highly contextual (Cullen et al., 2000). People support seemingly contradictory policies, show important gaps in knowledge, and can shift opinions based on information or framing (Garland, 2021). Public punitiveness is influenced by crime type and beliefs in the purpose of punishment, but also by research artifacts, like question framing and specific vs. general topics (see Indermaur et al., 2012).

Most relevant for the question of whether criminological knowledge may sway preferences for stronger punishment among the general public is a body of empirical work on how information provision and education may shape punitive attitudes. These studies look more broadly at so-called “information effects”. The idea is that preferences for stronger punishment are often based on false information or popular sentiment, originating for example in media sensationalism, political framing or ideological distortions. Garland (2021) suggests that a key role for academics is to provide reliable information to counter such falsehoods. Scholars indeed have found that punitive attitudes consistently respond to education and information in that informed people are less punitive across a number of contexts, including even in their support for capital punishment (Pickett, 2019). So far, this body of work has come to these findings by providing the public with information on topics like sentencing guidelines (Grimmelikhuijsen & van den Bos, 2021; Roberts et al., 2012), costs of incarceration and rates of re-offending (Gottlieb, 2017; Vuk et al., 2020), crime trends (Shi, 2021), false convictions (Bobo & Johnson, 2004; Norris & Mullinix, 2020; Wu, 2021), global trends in death penalty abolition (LaChappelle, 2014; Liang et al., 2019), psychological and physical effects of capital punishment on offenders (Sarat & Vidmar, 1976), and general school education about capital punishment (Bohm, 1989; Bohm et al., 1991; Cochran et al., 2006; Harmon et al., 2022; Kennedy‐Kollar & Mandery, 2010; Lee et al., 2014).

As this overview demonstrates, work on information effects generally has not looked at criminological evidence about the deterrent effect of punishment, and the way that this may impact people’s punishment preferences. There are, however, a few studies that have focused on related questions. Several studies have sought to test Justice Marshall’s (second) hypothesis, which holds that people will be less likely to support the death penalty if they are provided with accurate information about its use and effectiveness (Cochran, 2017; Sarat & Vidmar, 1976). Some of these studies also looked directly at the question how information about the deterrent effect of the death penalty may impact public support for capital punishment (Lambert et al., 2011; Lord et al., 1979; Sarat & Vidmar, 1976; Watamura et al., 2023). Sarat et al. (1976), studying a sample of 200 adults in Massachusetts, found that presenting scientific information about the deterrent effect of the death penalty reduced support for capital punishment, especially amongst people already opposed or only moderately supporting it. Similar findings were observed by Lambert et al. (2011) in a study amongst 700 college students. Conversely, Lord et al. (1979) found that such information increased polarization, such that scientific information increased partisans’ support for (or opposition against) capital punishment. Finally, a recent study conducted in Japan did not find that scientific evidence about the deterrent effect of the death penalty affected support for capital punishment (Watamura et al., 2023).

In sum, prior research generally suggests that providing scientific information to members of the general public can impact their punishment preferences. Moreover, some studies show that scientific information about the deterrent effect of the death penalty can influence public support for capital punishment. Yet, existing research provides little insight into how criminological evidence about the deterrent effect of punishment may impact preferences for the use of other forms of severe sanctions, such as harsher prison sentences. This is a vital question as prison sentences are far more common than capital punishment—even in the case of homicide, where the average sentence in the US is about 23 years (US Sentencing Commission, 2023). In light of the continuing trend toward more punitive criminal justice policy approaches (Dunbar, 2022), there is an urgent need to understand how presenting criminological evidence about deterrence can impact preferences for stronger punishment in members of the general public.

A recent study by Kuiper et al. (2023) provides preliminary insight into this question. It examined how science about the deterrent effect of punishment can affect punishment preferences among the general public with regard to prison sentences for violent crime. To do so, the study placed participants in the position to make (hypothetical) policy decisions and presented them with vignettes outlining a recent increase in crime within their jurisdiction. The study measured their preferences for increasing punishment in order to deter such offenses. Participants who were given scientific criminological evidence about the lack of conclusive evidence that stricter punishment deters violent crime were significantly less inclined to subsequently double the length of prison sentences, compared to a control group that received no scientific information. Furthermore, this effect was largely explained by reductions in the perceived efficacy of prison sentences as a means of deterring crime. This suggests that, on the face of it, simply providing appropriate scientific evidence can reduce public preferences for stronger punishment and bring their punishment preferences more in line with what science shows.

While these preliminary findings are promising, demonstrating the potential of criminological findings to bring public punishment preferences more in line with scientific evidence, there are many other factors within criminal justice settings that may modulate its effect. One factor is of particular importance for this question: the crime type. Kuiper et al. (2023) only investigated a single type of crime—burglary, a comparatively low seriousness offence. We know, however, that more serious crimes tend to evoke stronger punitive responses (Cochran et al., 2021) and tend to drive arguments for more severe punitive policy responses (Jennings et al., 2017). Yet, this is also where the evidence for stronger punishments as a deterrent is especially limited (Piquero et al., 2011). Therefore, to understand the value of criminological evidence as tool to produce public preferences about punishment more in line with science—a crucial question is whether knowledge of this evidence has equal impact regardless of the crime. That is, are punishment preferences regarding more serious crimes similarly impacted by exposure to scientific evidence as comparatively less serious crimes?

Moreover, Kuiper et al. (2023) also did not look at the psychology of how exactly scientific information comes to shape public punishment preferences. This is a vital issue as it may help us to understand what individual thought processes are at play when people process criminological scientific information in forming their punishment preferences. We know that how people process information about different crimes (i.e. attributions of cause, emotional reactions) as well as how they reflect on these attitudes and reactions can influence their punitiveness (Gromet & Darley, 2009; Persak, 2019; Petrocelli & Dowd, 2009). We also know that negative emotions (Hartnagel & Templeton, 2012; Johnson, 2009), views about redeemability (Burton et al., 2020), and general cognitive style (Sargent, 2004) play relevant roles in punishment attitudes. But we know yet little of how cognitive and psychological processes may explain the impact of criminological evidence on public punishment preferences, and the way that crime type may modulate this.

### Crime type and the impact of scientific evidence on public punishment preferences

What constitutes crime seriousness can differ based on subjective (e.g. perceived wrongfulness and harmfulness) and objective assessments (e.g. frequency and physical/economic impact, Michel, 2016). However, various crimes, particularly those involving physical and sexual violence, are generally perceived, and treated as more serious than non-violent or white-collar crimes—and these assessments are generally consistent across people (Borg et al., 2023). In turn, these differences in seriousness are reflected in different punishment preferences. More serious crimes are linked to increased willingness to punish and the severity of punishments made by individuals, increased punitive attitudes in the general public, and more punitive real-world sentencing outcomes (Adriaenssen et al., 2020; Apel, 2022; Atkin-Plunk, 2020; Michel, 2016; Payne et al., 2004). Crime type can also alter attitudes towards the goals of punishment (i.e. the purpose of sentencing). For example, Spiranovic et al. (2012) demonstrated that people tend to see deterrence as a more important reason for punishment for more serious offences (e.g. violent offending). Meaning, while presenting science may reduce the perceived importance of punishment for relatively less serious crimes—as found by Kuiper et al. (2023)—this may not hold when the crimes are more serious.

### Psychological and cognitive processes in the impact of crime type on punishment preferences

Research shows that psychological and cognitive processes have an effect on punitive attitudes (e.g., Indermaur et al., 2012; Krueger & Hoffman, 2016; Payne et al., 2004; Plantz et al., 2023; Sargent, 2004; Spiranovic et al., 2012) and thus also may moderate or mediate the effect of science on preferences for stronger punishment. The most relevant of such factors include: negative emotions people may have or develop about crime, personal ideas people have about the redeemability of people who commit crime, and people’s general cognitive style. For these reasons, we expected that these processes might explain the impact of science on punishment preferences, and the impact of crime type upon this.

*Negative emotions* Negative emotional reactions to crime, such as anger and fear, have been linked to increased support for punishment (Hartnagel & Templeton, 2012; Johnson, 2009). The intensity of these emotions also tends to vary based on the type of crime committed (Armborst, 2017). Stronger negative emotional reactions to crime, both fear and anger, are related to higher punitive attitudes and higher levels of support for punishment and punitive policies (Drakulich & Baranauskas, 2021; Kury & Kuhlmann, 2014) and may even be a key factor in why exposure to evidence fails to change attitudes towards punishment (Loader, 2005). It is possible that people who have stronger negative emotional responses (anger/fear) about crime will be less receptive to scientific evidence when making punishment decisions. Additionally, more serious types of crime may elicit stronger negative responses which may further impact receptiveness to such science.

*Redeemability* Support for punitive criminal justice tends to be higher if crime is attributed to internal (dispositional) factors like personal choice and lower when attributed to external (situational) factors such as socio-economics or context (Pickett & Baker, 2014; Updegrove et al., 2021). These attributions inform people’s perceptions of blameworthiness—which impacts intentions to punish (Cushman, 2008). For example, people who see crime as more situationally driven may be less likely to view a criminal as blameworthy or ‘at fault’. A final dimension is how stable these attributions are, that is to what extent people see factors that influence crime (dispositional vs. situational) as static over time (Maruna & King, 2009). These attributions inform beliefs of ‘redeemability’ about people who commit crime, which has been highlighted as a potential factor in explaining punitive decision making and specifically within the context of policy choices. More specifically, people are less likely to punish and more likely to support rehabilitative efforts if they display higher beliefs in redeemability (Burton et al., 2020). This makes it an interesting variable for this study, as people may perceive certain more serious crimes (and their context) as ‘less redeemable’ which may reduce how receptive they are to evidence that contradicts their intuition to punish (such as relevant scientific evidence).

*Cognitive style* Finally, the impact of science on punishment preferences may be further explained by elements of cognitive style, for example cognitive reflection and need for cognition (NFC). Cognitive reflection relates specifically to people’s ability or tendency to rely on intuitive vs. reflective responses in decisions (i.e. following ‘gut instinct’ vs. deliberating, Levy, 2023; Reyna et al., 2015), and has been shown to correlate with important predictors of punitiveness such as social and political conservatism (Deppe et al., 2015). Higher NFC is linked to greater engagement in effortful deliberation, avoidance of heuristics and biases, and preferences for more complicated explanations of human behavior (Tam et al., 2008). Higher NFC has been specifically linked to lower support for punishment, largely explained by an increased tendency towards more complex situational and environmental attributions of crime (Sargent, 2004) or counterfactual thinking (Petrocelli & Dowd, 2009). Therefore, people who demonstrate more reflective thinking styles and higher NFC may be more receptive to scientific evidence even when responding to more serious types of crime. That is, such people may be more likely to reflect on the evidence presented regardless of more intuitive instincts to punish more serious types of crime.

### The present study

The purpose of the present study is to investigate the role crime type and underlying psychological and cognitive factors play in the way that scientific evidence about deterrence affects public punishment preferences in relation to prison sentences for violent crime. For this purpose, this study aims to first replicate the findings of Kuiper et al. (2023), that exposure to scientific evidence will decrease preferences for stronger punishment. More specifically, we expect that when presented with the opportunity to increase prison sentences to deter offending, participants who are exposed to scientific evidence will be significantly less inclined to do so compared to those who are not (H1). And, as in Kuiper et al. (2023), that this relationship will be mediated by perceived effectiveness of such punishment—such that exposure to scientific evidence will reduce perceptions of severe punishments as an effective intervention, and thereby reduce preferences for stronger punishment (H2). Secondly, this study aims to extend on this replication by including two additional types of crime, to examine whether crime type (i.e., more/less serious) impacts participants’ punishment preferences, and the impact of scientific evidence upon this. Specifically, we expect that participants who are exposed to more serious types of crime will be significantly more likely to increase punishment than participants who are exposed to less serious types of crime (H3). We further hypothesize that the effect of science will be moderated by crime type with science having a weaker impact for more serious types of crime (H4). Third and finally, our study will exploratively assess how relevant psychological and cognitive variables (perceived seriousness, negative emotional response, redeemability, need for cognition (NFC), and cognitive reflection) may moderate or mediate the effect of scientific evidence on punishment preferences, and the impact of crime type upon this.

## Method

### Design

This study was an online experiment using a vignette study paradigm, and a 2 (Science vs. No Science) × 3 (Crime Type: Low, Medium, High Seriousness) between-subjects design. This resulted in a total of 6 conditions (see Table 1). The experimental procedure replicated the paradigm used in Kuiper et al. (2023) using the same base vignettes and same main dependent variable—adapted to allow for measuring the impact of crime type as a novel independent variable. This study included additional measures to explore novel variables of interest related to cognition (see Materials).

**Table 1:** Experimental conditions based on manipulated variables (crime type and science)

	Low crime seriousness (Burglary)	Medium crime seriousness (Armed Robbery)	High crime seriousness (Homicide)
Science	Condition 1	Condition 3	Condition 5
No science	Condition 2	Condition 4	Condition 6

### Participants

Participants were recruited via the online platform Prolific Academic (prolific.co). Based on a priori power analysis using G*Power (Faul et al., 2007) 330 participants was set as recruitment target.^1^ Additional inclusion criteria were that all participants were: (a) at least 18 years old; (b) English speaking; (c) currently living in the United States; and (d) had not participated in previous pilots/studies in the related research program.^2^ In total, 341 participants began the study and were randomly assigned to one of six conditions. However, seven participants were excluded for not finishing the study and were automatically replaced; a further four were excluded for failing one or more of the attention or manipulation checks.^3^ This resulted in a final sample of 330 participants^4^ (43.9% identifying as female) whose ages ranged from 18 to 76 years old (*M* = 35.75, *SD* = 12.79). Descriptive statistics for participants by condition are displayed in Table 2. Ethics approval was obtained as part of a wider research project in May 2020 from the University of Amsterdam Law School Ethics Review Board. All participants provided informed consent via a consent form prior to beginning the experiment. Participants were informed that their involvement was entirely voluntary and that they could withdraw from the study at any time. Participants were paid £14.69 per hour for their participation, with an average time of 8.09 min.

**Table 2:** Descriptive statistics by condition

	Condition					
	1	2	3	4	5	6
Variable^a^﻿	Burglary + Science	Burglary	Armed Robbery + Science	Armed Robbery	Homicide + Science	Homicide
*n*	53	56	55	52	57	57
Age (*M*(*SD*))	33.75 (10.84)	34.34 (11.02)	35.60 (12.55)	37.90 (14.64)	36.52 (12.76)	36.36 (14.01)
Trust in Science (*M*(*SD*))	4.17 (0.70)	3.93 (0.96)	3.94 (0.85)	3.99 (0.79)	3.87 (0.87)	4.13 (0.71)
Punitiveness (*M*(*SD*))	2.80 (0.98)	3.01 (0.96)	3.04 (0.87)	3.09 (1.15)	3.04 (1.02)	3.04 (0.85)
Need for Cognition *(M(SD))*	3.70 (0.90)	3.58 (0.82)	3.44 (0.82)	3.66 (0.89)	3.51 (0.99)	3.65 (0.93)
Cognitive Reflection *(M(SD))*	2.60 (1.12)	2.50 (0.99)	2.25(1.14)	2.32 (1.18)	2.65 (1.03)	2.30 (1.23)
Gender (%)						
Female	41.5	42.9	45.5	38.5	45.6	49.1
Male	58.5	57.1	52.7	55.8	45.6	50.9
Other/non-binary	0.0	0.0	1.8	5.8	8.8	0.0
Education (%)						
Some high school	0.0	0.0	0.0	0.0	0.0	1.8
High school diploma	13.2	161	9.1	19.2	12.3	12.3
Some college, no degree	22.6	16.1	18.2	13.5	15.8	22.1
Associates/technical degree	1.9	14	10.9	7.7	5.3	8.8
Bachelor’s degree	47.2	35.7	49.1	46.2	57.9	29.8
Graduate or Professional degree	15.1	17.9	12.7	13.5	7.0	26.3
Political orientation (%)						
Very liberal	11.3	16.1	29.1	26.9	22.8	19.3
Liberal	50.9	28.6	36.4	25.0	33.3	38.6
Moderate	24.5	48.2	14.5	30.8	29.8	29.8
Conservative	11.3	5.4	10.9	13.5	14.0	10.5
Very conservative	1.9	1.8	9.1	3.8	0.0	1.8
Experience (%)						
Social science	22.6	23.2	14.5	11.5	15.8	17.3
Law	9.4	8.9	5.5	5.8	1.8	5.3
Justice Policy & Admin	5.7	12.5	7.3	7.7	3.5	5.3

^a^No significant between-group differences were found on any of these measures

### Materials

All presentation of experimental materials and data collection occurred online using the platform Qualtrics.^5^

### Vignettes

*Crime vignettes* To assess their punishment preferences, the study placed participants in the role of a hypothetical policy maker tasked with developing interventions to reduce crime (cf. Kuiper et al., 2023). Doing so enables their punishment preferences to be assessed in a more substantive and engaging setting than general, decontextualized questions about punishment (Gelb, 2006; Roberts et al., 2003; Simonson, 2011). All participants received a vignette that outlined their role, and which explained that recently there has been a wave of crime within their jurisdiction. The type of crime described was one of three options: burglary,^6^ armed robbery, or homicide. The vignettes were identical apart from the description of the crime. Participants were informed that as policy makers, they were responsible for reducing crime in their jurisdiction but that due to funding limitations, they were unable to increase police resources. However, they were informed that they had the authority to double the level of punishment (prison sentences) for the crime in question.^7^ By making this hypothetical decision, the vignette measured participants’ preferences for stronger punishment as a means to deter offending. See Supplementary Material for full vignette.

*Science vignette* Participants in all the science conditions (see Table 1) received the science manipulation vignette which provided a short summary of key findings from criminology on deterrence theory and the severity and certainty of punishment. More specifically, this summary highlighted that there is no conclusive evidence that increasing the severity of punishment deters future offenders, that certainty is more important than severity, and that—unless a certain threshold of certainty is met—punishment does not deter offending (Brown, 1978; Chamlin, 1991; Helland & Tabarrok, 2007; Males & Macallair, 1999; Nagin, 2013a, 2013b; Shepherd, 2002; Zimring & Kamin, 2001). This vignette was previously piloted and used in previous studies in this research program.^8^ See Supplementary Material for full vignette.

### Questionnaire

This experiment included 18 main measured variables with data collected using a self-completed online questionnaire. There was one main dependent variable (*Punishment Preference*), one primary explanatory variable (*Perceived Effectiveness*) and 16 exploratory or control variables.^9^

*Punishment preference* To measure their punishment preferences, participants were presented with the decision whether to double the maximum sentence for the crime presented in the vignette in order to reduce such offenses in the future. A two-item measure was used for this purpose, adapted from Kuiper et al. (2023). Participants were asked to indicate to what extent they agreed with the following statements measured on a seven-point Likert scale from (1) Strongly Disagree to (7) Strongly Agree:

- “To effectively prevent [burglaries/armed robberies/homicide] in the future, I would double the average sentences for [burglaries/armed robberies/homicide] in my jurisdiction.”
- “To effectively reduce the rates of [burglaries/armed robberies/homicide] in the future, I would double the average sentences for [burglaries/armed robberies/homicide] in my jurisdiction.”

Correlational analysis showed a strong correlation between these items (*r* = 0.94*)*, hence, these were aggregated into a single scale measure: Punishment Preference.

*Perceived effectiveness of policy* Perceived effectiveness of punishment was measured by a single item, also adapted from Kuiper et al. (2023), measured on a seven-point Likert scale from (1) Strongly Disagree to (7) Strongly Agree: “I believe doubling sentences will be effective in reducing [burglaries/armed robberies/homicides].”

The following measures were included to address the third research aim (i.e. to identify additional mechanisms that may explain the relationship between science, crime type, and punitive policy decisions).

*Perceived seriousness of crime *Our measure for perceived seriousness used a two-item measure^10^ related to overall perceived harm severity and seriousness of crime, adapted for each crime type. These elements were chosen based on questions used in previous research (Adriaenssen et al., 2020; Michel, 2016; Stylianou, 2003). Items included:

- “Overall, how would you rate the level of harm caused by these [burglaries/armed robberies/homicides]? Measured on a five-point Likert scale from (1) No harm at all to (5) Very harmful.
- “Overall, how serious do you think these [burglaries/armed robberies/homicides] are?” Measured on a five-point Likert scale from (1) Not serious at all to (5) Very serious.

Correlational analysis showed a strong correlation between these items (*r* = 0.79*)*, hence, these were aggregated into a single scale measure: Perceived Seriousness.

*Negative emotional response to crime* Emotional response to crime, both individual response and perception of public response, was measured for two key negative emotions (fear and anger). Each was measured with one item on a five-point Likert scale from (1) Not [afraid/angry] at all to (5) Very [afraid/angry], resulting in a four-item measure (α = 0.81). Items were adapted from previous questions used by Hartnagel and Templeton (2012).

- “In general, how [angry/fearful] are you about the impact of [burglaries/armed robberies/homicides] in the US?”
- “In general, how [angry/fearful] do you believe the general public are about the impact of [burglaries/armed robberies/homicides] in the US?”

*Belief in redeemability of offenders* Perceived redeemability of offenders was measured using four items (α = 0.86) adapted from Burton et al. (2020), with each item being measured on a five-point Likert scale from (1) Strongly Disagree to (5) Strongly Agree. Each item in the scale was adapted for the relevant crime. Items three and four are reverse coded. Higher mean scores represented a greater belief in redeemability.

*Need for cognition* Need for Cognition (NFC) was measured using the six item NFC-6 (α = 0.90). The NFC-6 is an efficient scale used to measure people’s preference for engaging in effortful or deep thought (Lins de Holanda Coelho et al., 2020). Higher scores indicated a higher Need for Cognition.

*Cognitive reflection* Cognitive reflectiveness was measured using the Cognitive Reflection Test–2 (CRT-2, Thomson & Oppenheimer, 2016). The CRT is a commonly used measure of individuals tendencies towards reflection or intuition (i.e. ability to override intuitive or instinctive responses and make more reflective decisions). CRT-2 is a variation of the original CRT that has been developed to rely less on numerical ability. Scoring is calculated based on number of correctly answered items, with higher total scores reflecting a greater tendency for reflective thinking (i.e. ability to suppress intuitive responses).

The following variables were included to assess the effectiveness of our manipulations and check for individual differences between participants that could impact participants' sensitivity to the vignette or scientific information.

*Previous experience in social science, law, criminal justice policy or as a victim of crime* Participants were asked to indicate via a Yes/No item if they had experience in the form of study or training in social scientific research, law, or criminal justice policy/administration. Personal experience with violent crime was measured using one Yes/No item “Have you (or someone close to you) ever been victim of violent crime?”. If participants answered with yes, a follow-up question was asked whether this experience occurred in the last five years.

*Trust in science* Trust in science was measured using four items (α = 0.88) adapted from McCright et al. (2013) with each item using a five-point Likert scale from (1) Completely distrust to (5) Completely trust, with higher scores indicating more trust in science.

*Punitiveness* Apart from our specific measure assessing participants’ specific punishment preferences through their decision on doubling sentences, we also assessed their general level of punitiveness using five items (α = 0.94) on a five-point Likert scale from (1) Strongly disagree to (5) Strongly agree based on Mackey and Courtright (2000) and Spiranovic et al. (2012). Higher scores indicated greater punitiveness.

*Political orientation* Political orientation was measured using one item on a five-point Likert scale from (1) Very liberal to (5) Very conservative.

### Procedure

Participants first read the information sheet, provided their consent, and completed demographic measures. Each participant was then presented with the crime vignette for the condition they were assigned (conditions one and two = Burglary, conditions three and four = Armed Robbery, conditions five and six = Homicide). Participants who were in one of the science conditions (condition one, three and five) were next presented with the science vignette. Each vignette was presented for a minimum of 20 s during which the participant could not progress through the experiment. Immediately after presentation of the vignettes, participants were asked to complete the main dependent measures (Punishment Preference, Perceived Effectiveness). Participants then completed the post-experiment questionnaire containing the remaining measures listed in the Materials.

## Results

### Descriptive statistics

Table 3 displays Kendall’s Tau’s correlations^11^ and descriptive statistics (across conditions) for all main variables. Correlational values should be interpreted based on guidelines suggested by Gignac and Szodorai (2016), that is, 0.10–0.19 (weak), 0.20–29 (moderate), 0.30 or above (strong).^12^ There were significant strong positive correlations between Punishment Preference (i.e. likelihood of doubling sentences) and Perceived Effectiveness of policy (*τ*b = 0.73, *p* < 0.001), and Punitiveness (*τ*b = 0.44, *p* < 0.001); there were also moderate significant positive correlations with Negative Emotional Reaction (*τ*b = 0.26, *p* < 0.001), Perceived Seriousness (*τ*b = 0.14, *p* < 0.001), and Political Orientation (*τ*b = 0.25, *p* < 0.001)—with higher scores meaning more conservative tendencies. In addition, there were significant but weaker negative correlations between Punishment Preference and Trust in Science (*τ*b = − 0.20, *p* < 0.001), Belief in Redeemability (*τ*b = − 0.20, *p* < 0.001), Need for Cognition (*τ*b = − 0.10, *p* < 0.05), and Education Level (*τ*b = − 0.09, *p* < 0.05).

**Table 3:** Correlations and descriptive statistics for all variables^a^

	1	2	3	4	5	6	7	8	9	10	11	12	13	14	15	16	17
1. Punishment preference	–																
2. Age	0.02	–															
3. Gender	− 0.02	0.08	–														
4. Education level	− 0.09*	0.12**	0.05	–													
5. Exp. social science	− 0.07	− 0.8	− 0.03	0.17**	–												
6. Exp. law	− 0.02	0.05	0.00	0.10*	0.35**	–											
7. Exp. criminal justice policy	− 0.01	0.03	0.06	0.07	0.35**	0.63**	–										
8. Exp. victim	0.04	0.04	0.00	− 0.14**	0.08	0.17**	0.14*	–									
9. Exp. victim 5 years	− 0.07	− 0.08	0.04	0.00	0.13	− 0.04	0.03	–	–								
10. Political orientation	0.25**	− 0.09	0.02	− 0.05	0.04	− 0.01	0.03	0.02	0.02	–							
11. Trust in science	− 0.20**	0.05	− 0.03	0.07	− 0.01	0.1	0.03	0.04	0.04	− 0.33**	–						
12. NFC	− 0.10*	0.05	0.07	0.16**	0.12**	− 0.05	− 0.11*	− 0.10*	0.11	− 0.02	0.09*	–					
13. Cognitive reflection	− 0.05	− 0.13*	0.11*	0.06	− 0.03	− 0.02	− 0.02	0.03	0.03	− 0.06	− 0.05	0.10*	–				
14. Punitiveness	0.44**	− 0.04	− 0.02	− 0.04	− 0.17*	0.00	0.02	0.05	0.05	0.33**	− 0.23**	− 0.13*	− 0.09*	–−			
15. Perceived seriousness	0.14*	0.01	− 0.02	0.01	0.05	0.05	0.06	− 0.01	− 0.01	0.06	0.03	− 0.13**	0.00	0.11*	–		
16. Negative emotion	0.26*	0.13*	− 0.17*	− 0.05	0.02	− 0.01	0.02	− 0.03	− 0.03	0.11*	− 0.03	− 0.13**	− 0.15**	0.28**	0.29**	–	
17. Belief in Redeemability	− 0.21*	0.02	0.01	− 0.01	− 0.06	− 0.03	− 0.06	0.12	− 0.07	0.10*	0.15*	0.06	0.07	− 0.33**	− 0.32**	− 0.15*	**–**
18. Perceived policy effectiveness	0.73**	− 0.01	− 0.06	− 0.05	− 0.06	0.00	0.05	− 0.02	− 0.02	0.24**	− 0.12**	− 0.12**	− 0.06	0.45**	0.10*	0.28**	− 0.18**
M SD Scale Min/Max	3.96 1.99 1–7	35.7 12.7 –	0.45 0.40 0.1	4.3 1.3 1–7	0.17 0.40 0.1	0.06 0.24 0.1	0.07 0.26 0.1	0.26 0.44 0.1	0.41 0.50 0.1	2.4 1.03 0–4	4.00 0.79 1–5	3.6 0.92 1–5	2.4 1.11 0–4	3.00 0.97 1–5	4.22 0.85 1–5	3.48 0.86 1–5	3.49 1.88 1–7

n = 330. *p < 0.05 (2-tailed) **p < 0.01 (2-tailed). For Gender (0 = Male, 1 = Female): n = 321. For Experience measures (1 = Yes, 0 = No), Exp. Victim: n = 85; Exp. Victim 5 years: n = 35; Exp. Social Science: n = 57; Exp. Law: n = 20; Exp. Criminal Justice: n = 23)

### Main analysis

#### Impact of science and crime type on punishment preferences

Table 4 provides descriptive statistics for the main dependent and mediator variables by Science and Crime Type. The first aim of this study was to replicate the findings of Kuiper et al. (2023) that exposure to Science would significantly reduce preferences for stronger punishment (H1) and that this effect would be mediated by Perceived Effectiveness of increasing punishment (H2).

**Table 4:** Descriptive statistics for main dependent variable and potential mediator by science and crime type

Science	Crime type (M(SD))					
	Burglary		Armed Robbery		Homicide	
	Yes	No	Yes	No	Yes	No
Punishment Preference	2.66 (1.54)	4.67 (1.83)	3.15 (1.77)	5.01 (1.88)	3.37 (1.86)	4.88 (1.79)
Perceived Effectiveness	2.50 (1.46)	4.13 (1.08)	2.95 (1.63)	4.40 (1.92)	2.88 (1.75)	4.12 (1.80)

A two-way ANOVA^13^ was conducted to test for main effects and an interaction effect of Science and Crime Type on Punishment Preference. We found a significant main effect of Science, confirming H1. That is, participants who were presented with a summary of evidence outlining the limited effectiveness of harsher punishment deterring violent crime were significantly less inclined to double sentences than participants who did not receive this summary (*F*(2, 324) = 82.21, *p* < 0.001, η^2^ = 0.21).

**Fig. 1:**
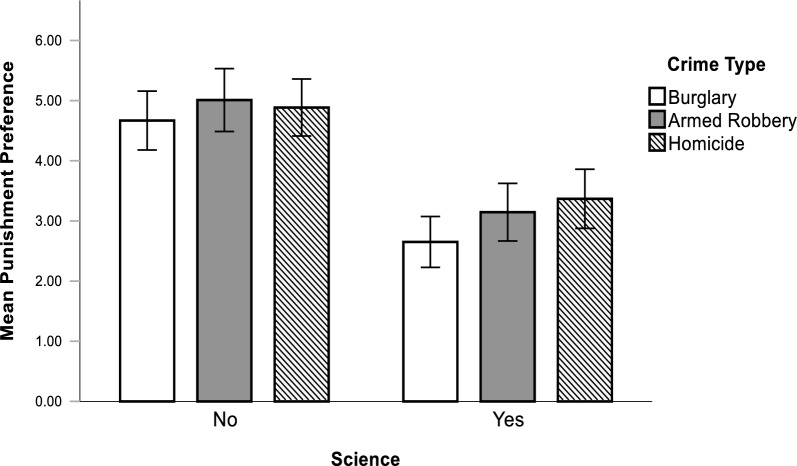
Mean punishment preference by exposure to science and crime type. Error bars indicate 95% CI

However, we found no support for H3 or H4 as there were no significant differences between participants’ likelihood to double sentences based on which type of crime they responded to, indicating no significant main effect of Crime Type on Punishment Preference (*F*(2, 324) = 2.78, *p* = 0.104, η^2^ = 0.014). Similarly, there was no significant interaction effect between Crime Type and Science^14^ (*F*(5, 324) = 0.581, *p* = 0.560 η^2^ = 0.004); see Fig. 1. As such, the effect of Science on participants likelihood to double sentences did not differ depending on the type of crime they responded to. Rather, Science significantly reduced preferences for stronger punishment no matter the severity of the type of crime.

#### Mediating role of perceived effectiveness

**Fig. 2:**
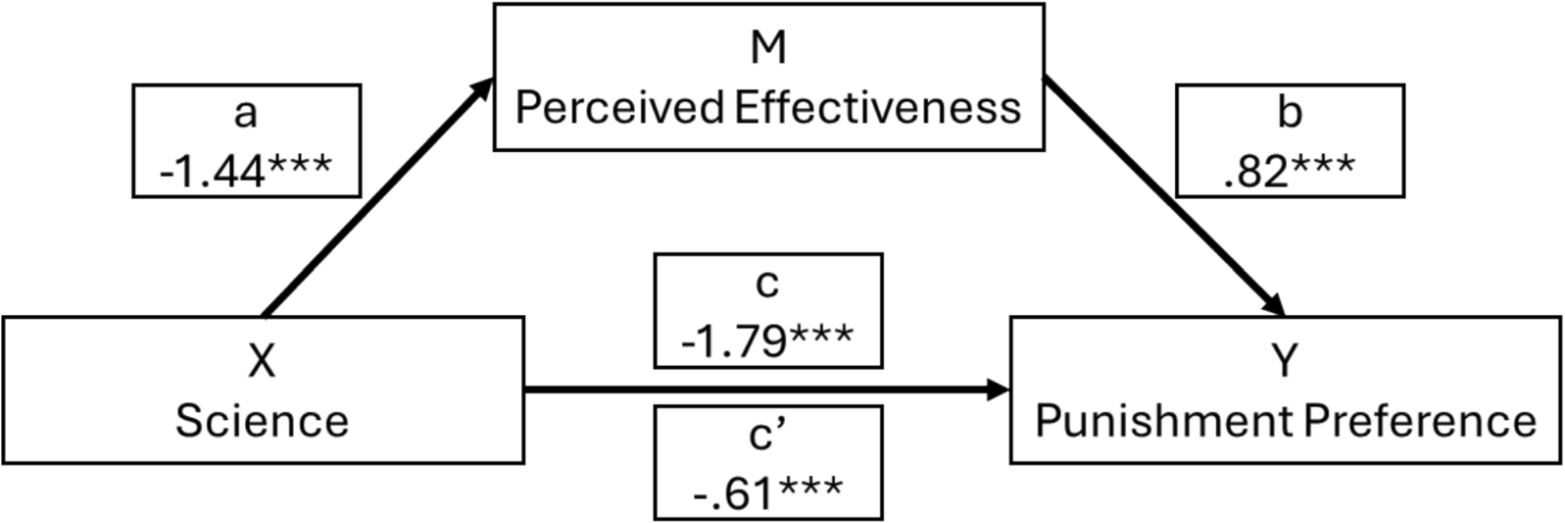
Mediation process for effect of science on punishment preference via perceived effectiveness ^a^c = total effect of science on punishment preference including perceived effectiveness; c’ = direct effect of science on punishment preference alone

To test H2 mediation analysis was conducted using Model 4 of the PROCESS Macro (Hayes, 2017) in SPSS,^15^ with Science as the independent variable, Punishment Preference the dependent variable and Perceived Effectiveness of the policy as the mediator. Results indicated that Science had a significant direct effect on Punishment Preference (*b* = − 0.61, 95% CI [− 1.81, − 1.06]), confirming H2. In addition, Science also had a significant direct negative effect on Perceived Effectiveness (*b* = − 1.44, 95% CI [− 1.81, − 1.06]), while greater Perceived Effectiveness had a significant positive effect on Punishment Preference (*b* = 0.82, 95% CI [0.75, 0.89]). And accordingly, we found a significant negative indirect effect of Science on Punishment Preference via Perceived Effectiveness (*b* = − 1.19, 95% CI [− 1.78, − 0.87]). What this indicates, as shown in Fig. 2, is that participants who were presented with scientific evidence perceived increasing punishment to deter crime as less effective than those who did not, and that people who saw increasing punishment as less effective showed lower preferences for increasing punishment.

### Moderation and mediation by psychological and cognitive variables

An additional aim of this study was to explore the role of relevant psychological and cognitive variables that could help explain the relationship between Science, Crime Type and Punishment Preference. These included Cognitive Style (NFC, Cognitive Reflection), Perceived Seriousness, Negative Emotion, and Belief in Redeemability. As we found no evidence of a direct relationship between these variables, that is, Crime Type did not appear to modify the effect of Science on Punishment Preference, nor impact Punishment Preference directly, we opted to examine relevant variables first as separate outcome measures—that may have been impacted by Science or Crime Type—and then secondly examine to see if relevant variables may have moderated or mediated the impacts of Science on Punishment Preference.

Additional individual two-way ANOVAs were conducted to test for main and interaction effects of Science and Crime Type on Perceived Seriousness, Negative Emotional response, and Belief in Redeemability (see Table 5 for descriptives).

**Table 5:** Descriptive statistics for exploratory dependent variables by science and crime type

Science	Crime type (M(SD))					
	Burglary		Armed Robbery		Homicide	
	Yes	No	Yes	No	Yes	No
Perceived Seriousness	3.62 (0.77)	3.74 (0.91)	4.09 (0.75)	3.95 (0.82)	4.90 (0.25)	4.95 (0.18)
Negative emotion	3.25 (0.86)	3.47 (0.84)	3.35 (0.79)	3.40 (0.91)	3.58 (0.88)	3.82 (0.84)
Belief in redeemability	3.56 (0.68)	3.73 (0.84)	3.30 (0.97)	3.44 (0.99)	2.61 (0.92)	2.67 (0.83)

#### Perceived seriousness

There were no significant interaction effects of Crime Type and Science on Perceived Seriousness (*F*(2, 324) = 1.04, *p* = 0.35), and no significant main effect of Science (*F*(1, 324) = 0.011, *p* = 0.92). However, there was a significant main effect of Crime Type (*F*(2, 324) = 102.14, *p* < 0.001, η^2^ = 0.39) indicating that participants’ perceived seriousness of crime differed significantly based on the type of crime they responded to. A post-hoc Tukey test^16^ revealed significant mean differences at the 0.05 level between all pairs (Burglary and Armed Robbery, Burglary and Homicide, and Armed Robbery and Homicide).

#### Negative emotional response

There were no significant interaction effects of Crime Type and Science on Negative Emotion (*F*(2, 324) = 0.44, *p* = 0.64), and no significant main effect of Science (*F*(1, 324) = 3.08, *p* = 0.08). There was a significant main effect of Crime Type (*F*(2, 324) = 5.68 *p* = 0.004, η^2^ = 0.03) indicating that participants’ negative emotional reactions differed significantly based on the type of crime they responded to. A post-hoc Tukey test revealed significant mean differences, at the 0.05 level, between Burglary and Homicide and Armed Robbery and Homicide, but no significant differences between Burglary and Armed Robbery.

#### Belief in redeemability

There were no significant interaction effects of Crime Type and Science on Belief in Redeemability (*F*(2, 324) = 0.11, *p* = 0.90), and no significant main effect of Science (*F*(1, 324) = 1.63, *p* = 0.20). There was a significant main effect of Crime Type (*F*(2, 324) = 39.19, *p* < 0.001, η^2^ = 0.19) indicating that participants’ Belief in Redeemability of people who commit crime differed significantly based on the type of crime they responded to. A post-hoc Tukey test revealed significant mean differences, at the 0.05 level, between Burglary and Homicide and Armed Robbery and Homicide, but no significant differences between Burglary and Armed Robbery.

#### Cognitive style

Exploratory analysis next examined the role that cognitive style measures (NFC and Cognitive Reflection) played in the relationship between Science and Punishment Preference. Based on prior research it was possible that participants who measured higher on NFC or Cognitive Reflection would be more inclined to utilize scientific evidence and less likely to show preferences for stronger punishment. We therefore ran a moderation analysis using Model 1 of the PROCESS Macro (Hayes, 2017) in SPSS to test if the effect of Science on Punishment Preference changed based on differences in participants measures for NFC or Cognitive Reflection. The results revealed no significant moderating impact of either NFC (*b* = 0.01, 95% CI [− 0.41, 0.43) or Cognitive Reflection on the relationship between Science and Punishment Preference (*b* = − 0.03*,* 95% CI [− 0.37, 0.32]). As such, we found no evidence that any of the cognitive style measures mediated or moderated the effect of Science on Punishment Preference.

## Discussion

The present study sought to understand whether presenting science about the deterrent effect of incarceration on violent crime has an effect on public punishment preferences. In particular, it sought to understand whether the effect of such science varies in light of crime seriousness and psychological and cognitive factors.

### Crime type and punitive policy decisions

Our results show that participants presented with scientific evidence about deterrence theory show significantly lower preference for stronger punishment. This effect can be, in large part, explained by a reduction in the perceived effectiveness of increasing punishment as a means to reduce crime. Further, we find that this is true even when the decision context involves more serious crimes—which previous research suggest increase punitive sentiments (Apel, 2022; Atkin-Plunk, 2020; Michel, 2016; Payne et al., 2004). In fact, our results specifically show that even though more serious crimes, particularly homicide, are viewed as less redeemable and generate more negative emotional responses—exposure to scientific evidence remains effective in reducing preferences for stronger punishments. This result replicates, and extends on, the findings of Kuiper et al. (2023) and underlines how providing the public with criminological evidence can reduce preferences for stronger punishment, and bring public punishment preferences more in line with scientific evidence.

Existing research highlights that different types of crime generate different punitive responses (Apel, 2022; Atkin-Plunk, 2020; Michel, 2016; Payne et al., 2004) as well as eliciting different emotional and cognitive responses—including perceptions of seriousness, and attributions of redeemability and blameworthiness—which may underly these punitive responses (Burton et al., 2020; Hartnagel & Templeton, 2012; Persak, 2019). Our results were only somewhat consistent with this. While more serious crimes (e.g., homicide) were perceived as more serious, elicited stronger negative emotional responses, and were seen as less redeemable, this had no significant effect on punishment preferences. It is possible that the observed differences in punishment preferences between the different crime types would reach significance in a higher-powered study. However, this would not alter the main conclusion of the present research, namely that scientific information reduces preferences for stronger punishment regardless of crime type. The observed effect sizes provide no indication that the impact of the science is reduced for the more serious crime types.

Why were no differences in punishment preferences observed between crime types? On one hand, this could indicate that people are not heavily influenced by their emotions and perceptions of particular crimes when considering their preferences for punishment in hypothetical policy contexts (i.e. at a population level)—which may differ to general punitiveness (e.g. attitudes towards punishment) or punishment preferences for individual cases of crime. However, this explanation alone is unconvincing given the biases that exist about certain crimes (both on an individual and societal level), and the link to subsequent punitiveness (Borg et al., 2023). As such, other explanations should be considered.

The lack of effect from crime type could reflect that people’s punishment preferences between crimes rely (in some part) on relative comparison. In the current study, participants’ punishment preferences were oriented on a single type of crime in isolation. It may be that people see any type of crime as severe enough to warrant attempts to reduce it (i.e. by increasing punishment), but that differences between particular crimes are only revealed when considering a variety of crimes concurrently. When considering sanctions for crimes in comparison to others, the relative seriousness and related emotional or cognitive impacts may become more relevant through direct comparison (e.g., one’s judgement about the seriousness of burglary in isolation may be relatively high, but may reduce when asked to concurrently consider the seriousness of homicide), and this may translate into different punishment preferences. Future research may build on the present findings to further explore these possibilities.

### Psychological factors, science, and punishment preferences

A secondary aim of this study was to examine whether psychological and cognitive factors might explain or shape the effects of science on punishment preferences, both in terms of how people think in response to crime as well as how they think more broadly (i.e. Cognitive Style). Our findings showed that while certain psychological and cognitive variables were related to punishment preferences, the effect of science did not depend on either of our measures for individuals' cognition. Specifically, our measures of Cognitive Style (Need for Cognition and Cognitive Reflection) did not moderate the relationship between exposure to scientific evidence and punishment preference. This could suggest that when scientific evidence is summarized in a clear and concise manner (at least in instances where this is possible), this can seemingly be persuasive both for individuals who engage in deep or in shallow processing.

One explanation for our results about psychological and cognitive variables may be that participants in our study based their punishment preferences on reflective processing and evaluation of the evidence presented to them. In our study participants without access to contrary scientific evidence showed a clear preference for stronger punishment (regardless of the crime), perhaps reflecting existing intuitive punitive tendencies towards crime (Darley & Alter, 2013). Whereas those provided with contrary scientific evidence appear to have adjusted their preferences to reflect a new more educated position—perhaps indicating a richer reflective process of weighing the evidence alongside existing beliefs and attitudes. This could lead to the conclusion that simply by ensuring the public have access to relevant and timely evidence they may develop more evidence-aligned punishment preferences. While a promising finding, evidence from cognitive science and behavioral economics show how easily people fail to engage in such reflexive processing in the real world (i.e. Kahneman, 2011; Tversky & Kahneman, 1974). This does not mean that reflective processing is not possible, but suggests that other variables (e.g. time pressures, political factors) that we did not include in our paradigm may disrupt this. Further discussion on these factors is included in the limitations below.

### Implications for punishment preferences and information

The present study shows further evidence that providing the public with information alters their punishment preferences, in making them less inclined to support strict punishment. Most existing studies in this realm have not focused on the provision of scientific information, but on information on issues such as crime trends, wrongful convictions, and sentencing processes (e.g. Bobo & Johnson, 2004; Bohm, 1989; Gotlieb et al., 2017; Grimmelikhuijsen & van den Bos, 2021; LaChappelle, 2014; Liang et al., 2019; Norris & Mullinix, 2020; Roberts et al., 2012; Shi, 2021; Vuk et al., 2020; Wu, 2021). The few studies that did do so (Lambert & Clarke, 2001; Lambert et al., 2011; Sarat & Vidmar, 1976; Watamura et al., 2023), did so testing Justice Marshall’s second hypothesis that informing the general public on the deterrent effect of capital punishment would reduce their support for the death penalty (Cochran, 2017). The present research clearly shows (in line with Kuiper et al. (2023)) that scientific information about deterrence should be a key element in theoretical and practical approaches to address public punitiveness. It moves our understanding of this finding beyond Kuiper et al. (2023) by showing that this approach can work for a broad range of violent crimes, even ones that elicit strong emotional responses, such as homicide. Moreover, it also advances our understanding of these processes beyond the preliminary findings of Kuiper et al. (2023) by demonstrating that, at least in the conditions studied, cognitive and psychological processes do not necessarily hamper successful transmission of science into public understanding and their application into more scientifically informed punishment preferences.

The study thus has vital implications for the study of punishment preferences and punitiveness and the information hypothesis that is central to this (Garland, 2021; Pickett, 2019). It shows that this field must look further and deeper into the effects of providing the public with criminological scientific information. Moreover, it shows that the body of work that has looked at providing science about deterrence must look beyond Marshall’s original hypotheses about the death penalty, to assess its effects also for more commonly used forms of punishment such as imprisonment. Finally, it shows that translational criminology (Blomberg et al., 2022; Laub & Frisch, 2016; Nichols et al., 2019; Pesta et al., 2019)), which has sought to build evidence based criminal justice practice by enhancing exchanges between criminological science and practice, should also focus on scientific dissemination towards the general public. The present study points towards a collaboration between the fields of research on punishment preferences and translational criminology, to advance our understanding of how science can best be presented to truly make public punishment preferences more aligned with empirical insights.

### Limitations and future research

While our results provide valuable new insights into the processes through which scientific evidence can be successfully translated into more informed punishment preferences in the general public, there are a number of key limitations that impact the strength and generalizability of our conclusions.

A first set of limitations relate to the study design. Our measure for punishment preferences may have been insensitive to differences between crime types when measured as a between-subjects factor. To explore this further, future research could investigate crime type as a within-subjects factor by allowing participants to indicate punishment preferences for multiple crime types. This would make it possible to test if direct comparisons between crime types translate to different preferences for increasing punishment. Further, using measures that permit more relative changes in punishment preferences, such as for increasing or decreasing sentences (by half, quarter) for each crime type could also address this limitation. Additionally, exploring the impact of scientific evidence when considering a broader range of functions of punishment (e.g. deterrence, rehabilitation, prevention) may represent a useful adaptation of the present approach.

Furthermore, although we assessed participants’ preferences for increased punishment (i.e., doubling the existing sentences), our materials did not specify explicitly what exactly such doubling entailed. For the most serious crime studied here, homicide, this may well have meant that some participants may have had the false impression that average sentences for murder are already at a level (life without parole) where doubling would be meaningless. While this is not the case, and sentences for murder average 272 months in the US (US Sentencing Commission, 2023), our instructions did not specify this. Future research should examine whether the present findings replicate when current sentences (and the scope for doubling them) are made explicit. Also, such research should include dedicated (control) questions to verify that participants understood that doubling was meaningful.

A further limitation of our study design is its focus on violent offenses. Our research therefore does not inform how scientific evidence may impact punishment preferences for nonviolent offenses. We focused on violent offenses for two reasons: firstly, because these are politically the most salient, and secondly, because the scientific evidence that questions the deterrent effect of punishment principally relates to violent offenses, whereas for non-violent offenses, there is scientific evidence that punishment may deter (see Dölling et al., 2009). How such findings may impact punishment preferences for nonviolent offenses (such as public order, drug, and property offenses) would be an important avenue for future research.

A final limitation concerning our study design is that by focusing on the deterrent effect of punishment on violent crime, we looked at a question for which the scientific evidence has been relatively consistent and conclusive (see Dölling et al., 2009; Nagin, 2013a). In contrast, scientific evidence about other effects of punishment (such as incapacitation, criminogenic effects, or adaptation) are more ambiguous, with mixed or contradictory findings (van Rooij et al., 2024). Future research could examine if the effect of science on punishment preferences is maintained even when science is more ambiguous or nuanced.

A second set of limitations concerns the generalizability of our findings. This firstly concerns the nature of our sample. Although previous research has demonstrated that Prolific samples rate highly in terms of data quality and diversity compared to other online research panels and student samples (see e.g. Douglas et al., 2023; Pe'er et al., 2017, 2021; Stagnaro et al., 2024; Uittenhove et al., 2023), the resulting samples nevertheless do not necessarily reflect the general population as a whole. Illustrating this point, our current sample offered good variability in terms of age and gender, but was relatively progressive and highly educated (while still offering considerable variation in these characteristics). It would be valuable for future research to examine how the present findings may translate to other samples, particularly among more conservative and less educated individuals. Second, the present study only assesses the immediate effects of science on punishment preferences. We do not know to what extent reading science affects such preferences more broadly over the longer term, nor what strategies can best be used to disseminate science to create a longer lasting effect. Future studies could use longitudinal designs to assess these effects over a longer term and also test strategies to enhance the endurance of scientific dissemination. Third, the present study does not look directly at how science affects public electoral decisions. Such attitudes have the most direct influence on policy and law through political actions citizens take when voting in elections or referenda on specific criminal justice issues (Shapiro, 2011). Furthermore, public officials tend to follow the public’s sentiment about issues such as crime and punishment (Pickett, 2019). Future studies could adapt the present study paradigms to look more directly at the role science provision may play in such electoral decision making.

## Conclusion

This study provides compelling findings that further clarify the circumstances required for criminological evidence to be successfully utilized to shape the punishment preferences of the general public. This study demonstrated that presenting people with timely and accessible scientific evidence can lead to more evidence aligned punishment preferences—which are robust to contextual factors such as the seriousness of crime and related impact on individual emotional and cognitive response. This highlights a number of valuable insights for criminology as it seeks to translate its empirical evidence into real-world practical contributions. It suggests that public opinion about punishment may be brought more into line with scientific evidence by improving access to consumable, reliable evidence. By enabling more scientifically-informed punishment preferences in the general public, criminology may also come to impact real world criminal justice policy, and counter the continuing reliance on punitive approaches existing there. It is our hope that future research will build upon these findings to help further unlock the potential of criminological science for these purposes.

## Supplementary Information

**Supplementary file 1**. Appendix.

## Data Availability

For a complete overview of all survey materials. Items and data please see: https://osf.io/k4ny7/.

## Footnotes

1 Power analysis was based on the need to detect a significant effect in a 2 (Science exposure: yes or no) × 3 (Crime Type: low, medium, high seriousness) between-subjects ANOVA, assuming an effect size Cohen’s f = .25, power of .95, and α = 0.05. This indicated a sample of 323, in an effort to ensure that groups would be equal in size 330 participants were recruited. Parameters for this power analysis were based on those used by Kuiper et al. (2023) for a similar experimental paradigm and procedure.

2 The Prolific platform allows researchers to pre-screen participant accounts that have participated in other studies the researchers have run through the Prolific platform. See: https://researcher-help.prolific.com/hc/en-gb/articles/360009094374-How-can-I-prevent-specific-participants-from-accessing-my-study. For this study, this meant that participants who had previously participated in Kuiper et al. (2023) or any of its preceding pilot studies, were ineligible to take part.

3 Participants received a minimum of two attention checks. Participants in the science conditions received an additional multi-choice memory check, asking them to confirm what the research topic they read was about.

4 Due to the random allocation and replacement procedure of participants final groups sizes were not equal, however these were small differences and largely equal for the main independent variables.

5 For a complete overview of all survey materials. Items and data please see: https://osf.io/k4ny7/?view_only=f8225086a0f941bf80cf3305893fca1e.

6 This vignette was identical to the vignette used in prior studies within this research programme, see: https://osf.io/9g6km/?view_only=799954a0a1f44d578d916ca7fa00cf86.

7 Average sentences in the US for these crimes average 21 months for burglary (also including trespass), 111 months for robbery, and 272 months for homicide (US Sentencing Commission, 2023). As such, sentences for all three crimes could realistically be doubled.

8 Previous pilots tested different ways to present scientific information and did not find differences in participants’ perceptions of the presented science, subjective understanding, or objective understanding. See: https://osf.io/2vjtg/?view_only=1047b222191842a7b5f2a12ef9829801.

9 Item order was randomised for multi-item measures.

10 Factor analysis was conducted to check for validity of the following measures: Punishment Preference, Perceived Seriousness, Negative emotional response. In all cases a single factor was identified meaning and a single item was computed for each.

11 Nonparametric Kendall’s Tau correlations were chosen as there were a mix of continuous and categorical variables included with the analysis.

12 Note that due to the high number of variables (18) included in the correlational analysis there is an increased chance of false positives (Type 1 error). However, correcting for multiple comparisons was deemed too conservative as adjusting for the number of comparisons (152) would have made it overly difficult to detect any significant correlations.

13 SPSS 28.0 was the statistical software used. Tests for assumptions of Equality of Error Variances and Heteroskedasticity were not significant. Bonferroni corrections for multiple comparisons were made for all analyses.

14 As Crime Type had no significant moderating effect on the effect of Science, no further analysis were conducted to test the role of potential moderating effects of Need for Cognition or Cognitive Reflection.

15 For all tests we used bootstrapping procedures to test for significance, effects were calculated using 5000 bootstrapped samples and 95 percent confidence level.

16 Bonferonni adjustment used for multiple comparisons.
