# Supplementary material for: Can criminology sway the public? How empirical findings about deterrence affect public punishment preferences
Source: Crime Sci. 2024 Dec 18;13(1):43. doi: 10.1186/s40163-024-00240-8 (PMC11655585; doi:10.1186/s40163-024-00240-8)
Supplement: Supplementary file 1 — Appendix. [file 40163_2024_240_MOESM1_ESM.pdf]

Appendix A – Crime Case Vignettes

1. **Case vignette – Burglary – Low Seriousness (Condition 1, 2)**

In the next part of the study, you will read information about a crime case. **It is very important that you take your time when reading**. Afterwards, you will be asked several questions about the information.

Please read the following case carefully.

Imagine that you are tasked with developing policy interventions to try to reduce crime.

In your jurisdiction there has recently been a wave of home burglaries. In several neighbourhoods, thieves have broken into homes when no one was there, and each time they have stolen several valuable items.

It is your task to come up with an effective response to prevent this type of crime in the future. Currently, the chance of catching the perpetrators is low, as there is a shortage of available police officers. There are little resources at your disposal to increase the chance of catching the perpetrators, as you have no funds to spend on extra police officers or other measures. Thus, you are asked to prevent home burglaries without being able to increase the chance of arresting the perpetrators. You do, however, have the authority to change the level of punishment for committing this type of crime.

2. **Case vignette – Armed Robbery – Medium Seriousness (Condition 3, 4)**

In the next part of the study, you will read information about a crime case. **It is very important that you take your time when reading**. Afterwards, you will be asked several questions about the information. *Please read the following case carefully.*

Imagine that you are tasked with developing policy interventions to try to reduce crime.

In your jurisdiction there has recently been a wave of armed robberies. In several neighbourhoods there have been cases of armed robberies on the street, in shopping malls, and in other public spaces. While some individuals have received minor injuries, luckily no one was killed during any of these incidents.

It is your task to come up with an effective response to prevent this type of crime in the future. Currently, the chance of catching the perpetrators is low, as there is a shortage of available police officers. There are little resources at your disposal to increase the chance of catching the perpetrators, as you have no funds to spend on extra police officers or other measures. Thus, you are asked to prevent armed robberies without being able to increase the chance of arresting the perpetrators. You do, however, have the authority to change the level of punishment for committing this type of crime.

3. **Case vignette – Murder – High Seriousness (Condition 5, 6)**

In the next part of the study, you will read information about a crime case. **It is very important that you take your time when reading**. Afterwards, you will be asked several questions about the information. *Please read the following case carefully.*

Imagine that you are tasked with developing policy interventions to try to reduce crime.

In your jurisdiction there has recently been a wave of homicides. In several neighbourhoods, there have been incidents where people were killed at home, in the street, or in other public places. It is not yet clear if these cases are unconnected or linked.

It is your task to come up with an effective response to prevent this type of crime in the future. Currently, the chance of catching the perpetrators is low, as there is a shortage of available police officers. There are little resources at your disposal to increase the chance of catching the perpetrators, as you have no funds to spend on extra police officers or other measures. Thus, you are asked to prevent homicides without being able to increase the chance of arresting the perpetrators. You do, however, have the authority to change the level of punishment for committing this type of crime.

Appendix B – Science Vignette (for conditions 1, 3, 5) and Main Question

Next, you are asked to read a short piece of information that describes findings from scientific research. **It is very important that you take your time when reading** and try to remember the information.

Afterwards, you will be asked several questions about the information. We will also test your memory of the information.

<u>The following text describes what scientific evidence concludes about crime prevention. Please read it carefully.</u>

Decades of scientific research has shown that there is no conclusive evidence that more severe punishment helps to reduce crime (Helland & Tabarrok, 2007; Males & Macallair, 1999; Shepherd, 2022; Zimring & Kamin, 2001).

However, there is evidence that increases in police presence do reduces crime, as they increase the certainty that offenders are caught and receive punishment (Nagin, 2013a).

In general, science concludes that increasing punishment will only deter crime, if the chance of getting caught is sufficiently high (Nagin, 2013b). Only if there is a threshold level of certainty of getting caught and punished does stronger punishment start to prevent crime (Brown, 1978; Chamlin, 1991).

In conclusion, the scientific evidence does not provide proof that increasing punishment when there is insufficient police capacity will help to reduce crime.

Appendix C - Main Question

Remember that it is your task to reduce crime, and that there recently has been a [home burglaries/armed robbery/homicide]. In your job, you have the authority to make decisions about punishment in order to reduce crime.

Remember that there are no resources at your disposal to increase the chance of catching the perpetrators, as you have no money to spend on extra police officers or other measures.

Please indicate how much you agree or disagree with the following statements:

"To effectively prevent [home burglaries/armed robbery/homicide] in the future, I would double the average sentences for [burglary/armed robbery/homicide] in my jurisdiction."

"To effectively reduce rates of [home burglaries/armed robbery/homicide] in the future, I would double the average sentences for [burglary/armed robbery/homicide] in my jurisdiction."
